# Effects of Induced Endotoxaemia on Global Cardiovascular, Oxygenation and Haematologic Variables and the Integrity of the Endothelial Glycocalyx in the Horse

**DOI:** 10.1002/vms3.70458

**Published:** 2025-06-19

**Authors:** Stephan Neudeck, Philipp K. Sauter, Annette P. N. Kutter, Barbara Steblaj, Franz J. Söbbeler, Julia Reiners, Fritjof Freise, Alvaro. J. Gutiérrez Bautitsta, Sabine B. R. Kästner

**Affiliations:** ^1^ Clinic for Horses University of Veterinary Medicine Hannover, Foundation Hannover Germany; ^2^ Clinic for Small Animals University of Veterinary Medicine Hannover, Foundation Hannover Germany; ^3^ G. Pohl‐Boskamp GmbH & Co. KG Hohelockenstedt Germany; ^4^ Section of Anaesthesiology Vetsuisse Faculty University of Zurich Zurich Switzerland; ^5^ Tierärztliche Kinik für Kleintiere am Kaiserberg Duisburg Germany; ^6^ Department of Biometry Epidemiology and Information Processing University of Veterinary Medicine Hannover, Foundation Hannover Germany

**Keywords:** endotoxaemia, equine, glycocalyx, macrocirculation

## Abstract

**Background:**

Endotoxaemia is a significant cause of morbidity and mortality in equids due to perfusion impairment and possible destruction of the glycocalyx.

**Objectives:**

To evaluate our hypothesis that endotoxaemia induces changes in global cardiovascular and haematologic parameters and compromises glycocalyx integrity, evidenced by an early rise in plasma shedding products.

**Study design:**

In vivo experiments

**Methods:**

In a prospective, randomised, controlled experimental trial, endotoxaemia was induced with E. coli B55:O5 LPS 30 ng kg^−1^ over 30 min IV in six healthy adult horses ventilated with oxygen supplemented with isoflurane. Standard cardiovascular variables were recorded and calculated, and leucocyte counts, lactate, heparan sulphate and syndecan‐1 concentration were determined at baseline (B) before endotoxin and at 0, 30, 60 and 120 min after endotoxin. Data were analysed using mixed models and adjusted by Tukey‐Kramer (SAS Enterprise Guide Software 7.1).

**Results:**

After endotoxin (120 min), a significant increase (p ≤ 0.05) in cardiac index (43 ± 9 vs. 80 ± 15 mL kg^−1^ min^−1^, p < 0.01), in oxygen delivery index (8 ± 3 vs. 17 ± 4 mL min^−1^ kg^−1^, p <0.001), in pulse pressure variation (8 ± 3 vs. 17 ± 4, p < 0.01) and in lactate (1.55 ± 0,9 vs. 4.4 ± 0.52 mmol L^−1^, p < 0.0001) occurred with a decrease in systemic vascular resistance index (247 ± 87 vs. 83 ± 20 dynes s kg cm^−5^, p < 0.001), diastolic arterial blood pressure (69 ± 14 vs. 38 ± 5 mmHg; p < 0.001), and leucocyte counts (5.6 ± 1.3 vs. 1.5 ± 0.3 G l^−1^, p < 0.0001). No changes in the glycocalyx degradation products could be found.

**Conclusion:**

Short‐term experimental endotoxaemia under isoflurane induced anticipated cardiovascular changes but did not alter glycocalyx shedding products in this study.

## Graphical abstract

The current study demonstrated characteristic and well‐documented early hyperdynamic changes in cardiovascular parameters accompanied by concurrent hypotension, increased pulse pressure variation, leucopenia, and hyperlactatemia consistent with acute endotoxaemia. Notwithstanding, no consistent increase in glycocalyx‐shedding products was observed during this approximately 2.5 h period of endotoxaemia.

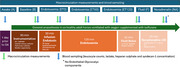

## 1 Introduction

Endotoxaemia is a significant cause of morbidity and mortality in equids (Moore and Barton 2003; Werners et al. 2005). Pathological endotoxaemia is present in various diseases, including colic, laminitis (Moore et al. 1979), peritonitis, metritis, neonatal sepsis, or pleuropneumonia (Shuster et al. 1997; Beeler‐Marfisi et al. 2010). In colic, mortality is closely related to the degree of endotoxaemia, and clinical and clinicopathologic signs of endotoxaemia can be used as prognostic indicators, correlating with increased mortality (Parry et al. 1983). Endotoxaemia can lead to a dysregulated immune response, restricted tissue perfusion, reduced oxygen exchange, and organ hypoxia. The endotoxaemia causes microcirculatory disturbances with consequent impairment of macrohaemodynamics and organ dysfunction (Morris 1991; Sauter et al. 2025; McCuskey et al. 1996). Clinical signs include hypotension, hypovolemia, coagulopathy, and multiple organ dysfunction (Werners et al. 2005). Initially, the systemic inflammatory reaction leads to vasodilation associated with decreased mean arterial pressure, reactive tachycardia, and increased cardiac index. These compensatory mechanisms induce an early hyperdynamic process (Burrows 1970). The release of vasoactive substances might lead to endothelial damage. This condition is accompanied by impaired oxygen utilisation and increasing blood lactate concentration (Moore et al. 1980). Later, this hyperdynamic phase can change into a hypodynamic phase with a characteristic decrease in mean arterial blood pressure, cardiac index and compensatory increase in peripheral resistance with a consecutive mismatch of oxygen delivery and consumption (Burrows 1971). Haematologically, a left shift of neutrophil granulocytes and leukopenia are prominent (Burrows 1970).

The endothelial glycocalyx forms the primary boundary between the endothelium and the circulating blood. This luminal layer lines the endothelium of all vessels. Proteoglycans, including heparan sulphate and syndecan‐1, glycoproteins, and glycolipids, form the membrane‐bound part of the glycocalyx. Proteoglycans are a component of the animal extracellular matrix and consist of a protein and one or more covalently bound carbohydrate groups, the glycosaminoglycans (GAGs). Among many functions of the endothelial glycocalyx, this structure is vital in maintaining and regulating vascular homeostasis (Uchimido et al. 2019), vascular permeability (Reitsma et al. 2007), and vascular mechanotransduction (Gaudette et al. 2020).

Loss or degradation of the endothelial glycocalyx affects inflammatory processes, coagulation, and vascular permeability. It has been shown that endotoxaemia and several other pathological conditions can cause damage to the endothelial glycocalyx (Gaudette et al. 2020). Postischemic organ damage, sepsis, inflammation, renal disease, diabetic vasculopathy, and atherosclerosis are discussed as causes in humans (Becker et al. 2015). The degradation is associated with wash‐off and leaking of some endothelial glycocalyx (EG) components into the blood. A G‐protein‐sensitive signalling pathway activates proteases or lyases during inflammation (Mulivor and Lipowsky 2004). Quantitative enzyme‐linked immune assays (ELISAs) can be used to measure shedding products of the deranged endothelial glycocalyx in blood. As a result of sepsis, plasma syndecan‐1 and heparan sulphate proteoglycan concentrations were increased in people (Anand et al. 2016; Saoraya et al. 2021) and dogs (Yini et al. 2015; Iba and Levy 2019). The severity of the disease, organ dysfunction, and mortality correlate with the level of the shedding product concentration in humans (Anand et al. 2016; Saoraya et al. 2021).

In horses, a recently published retrospective study, plasma syndecan‐1 concentrations were elevated in septic adult horses, indicating endothelial glycocalyx degradation. Here, elevated plasma syndecan‐1 levels were found for the first time in clinical equine patients (Hobbs et al. 2023). Since this study used clinically diseased horses, multiple factors could have influenced the integrity of the glycocalyx.

Fluid therapy plays a critical role in managing endotoxaemia. Determining whether a patient´s cardiac output will improve following fluid administration (fluid responsiveness) is essential to avoid under‐resuscitation or fluid overload, both of which can worsen outcomes (Araos et al. 2020). Dynamic indices such as pulse pressure variation (PPV) have emerged as promising tools for assessing fluid responsiveness, leveraging heart‐lung interactions during mechanical ventilation. While studied in humans and small animals (Ambrisko et al. 2012), the utility of PPV in horses, especially under endotoxemic conditions, remains poorly defined.

The goal of the current study was to describe changes in cardiovascular, haematological and endothelial glycocalyx degradation products in a group of six anaesthetised, clinically healthy horses after experimental induction of endotoxaemia and its treatment with fluids and vasopressors. We hypothesise, that the intravenous administration of the lipopolysaccharide Escherichia coli O55:B5 would lead to changes in global cardiovascular and haematologic variables and would affect the glycocalyx integrity detectable by an early increase in shedding products in the bloodstream.

## 2 Materials and Methods

### 2.1 Animals

A total of six horses of different breeds (5 Warmbloods and 1 Thoroughbred) and sexes (two geldings and four mares) were included in the study. The horses owned by the equine clinic of the University of Veterinary Medicine Hannover Foundation , were kept with access to pasture and fed with hay daily. The horses were placed in individual stalls one day before the studies. Food and water were not withheld before the experiment. The patients were considered healthy regarding cardiovascular and intestinal function based on clinical and haematological examination, though they had untreatable orthopaedic problems. The horses in this study were part of two concomitant studies that required median laparotomy (Sauter et al. 2025). After conclusion of the final study, the cadavers were used for anatomical and arthroscopic teaching purposes. Ethical review and approval were obtained from the Ethical Committee of Lower Saxony (approval number 33.19‐42502‐04‐20/3481).

### 2.2 Study Design

The study was designed as an experimental, terminal trial. The power analysis was based on syndecan‐1 values collected from dogs with induced septic shock, resulting in a size of six horses with a power of 80%, an alpha of 5% and an effect size of 1.7 (Yini et al. 2015). At the time of calculation, there were no syndecan values published for horses.

### 2.3 Instrumentation and Anaesthesia

A 12G catheter was placed aseptically after subcutaneous infiltration of lidocaine in the left jugular vein. All horses received a bolus of 5 µg kg^−1^ dexmedetomidine (Dexdomitor 0,5 mg/ml; Orion Pharma, Finland) intravenously (IV) for sedation. Additionally, two introducer ports (Exacta, 8.5 Fr; Argon Medical Devices Inc., IL, United States of America) were placed aseptically in the right jugular vein. The central venous and pulmonary catheters (Balloon wedge pressure catheter, 7 Fr, 160 cm; Arrow International Inc., NC, USA or TVEC catheter 7 Fr, 200 cm, Gaeltec, Scotland) were then inserted through the introducers in front of the right atrium and into a pulmonary artery, respectively, under the control of location‐specific pressure curves in the standing and sedated animal.

General anaesthesia was induced with 2.5 mg kg^−1^ ketamine IV (Narketan 100 mg/ml, Vetoquinol GmbH; Germany) and 0.05 mg kg^−1^ diazepam IV (Ziapam 5 mg/ml, Ecuphar; Germany). After induction, the horses were endotracheally intubated and placed in dorsal recumbency on a padded surgical table. A large animal ventilator (Vet. ‐Tec. Model JAVC 2000, J.D. Medical Distributing Company, state, United States of America) was connected, and intermittent positive pressure ventilation was initiated. Anaesthesia was maintained with isoflurane in 100% oxygen and was adjusted to effect (end‐expiratory value of 1.3 to 1.5 vol.%) to achieve an adequate aesthetic stage. The respiratory rate and peak inspiratory pressure were adjusted to keep the horses normocapnic with a targeted end‐tidal carbon dioxide partial pressure (ETCO_2_) of 35–45 mmHg. Lactated Ringer's solution was given with an infusion pump at a fixed rate of 5 mL kg^−1^ h^−1^. The horses received a constant rate infusion (CRI) of dobutamine 0.33 µg kg^−1^ h^−1^ IV by a syringe driver.

Arterial catheters (VenocanTM PLUS IV Catheter 22G.33 mm, KRUUSE A/S, Denmark) were placed in the facial and the transverse facial arteries in the anaesthetised animal. To determine cardiac output, the lithium dilution technique was performed as described elsewhere (Ambrisko et al. 2012) using the LIDCO system (LiDCOplus haemodynamic Monitor, LiDCO Ltd., Great Britain ). The arterial and central venous catheter ports were connected to fluid‐filled, low‐compliance extension lines of two pressure transducers (Argon Safedraw Transducer; Argon Medical Devices Inc., IL, United States of America), which were placed at the level of the scapulohumeral joint and zeroed to atmospheric pressure for blood pressure measurement.

### 2.4 Interventions

Endotoxaemia was induced IV by infusing 30 ng kg^−1^ of lipopolysaccharide (Escherichia coli B55:O5 LPS) (L2880‐10MG, Merck KGaA, Germany) in 1000 mL 0.9% saline over 30 min by an infusion pump, and measurements were performed every 30 min. Following a 120 min endotoxaemia period, a 10 mL kg^−1^ bolus of lactated Ringer's solution was administered over 20 min via an infusion pump. After the fluid bolus, noradrenaline was infused with the aim to normalise blood pressure with a target mean arterial blood pressure (MAP) of 80–90 mmHg. As a starting dose, 0.1 µg kg^−1^ min^−1^ noradrenaline (NA) was administered using a syringe pump. If no significant increase in MAP could be induced, the noradrenaline dosage was increased every 5 min up to 0.8 µg kg^−1^ min^−1^

### 2.5 Measurements

#### 2.5.1 Cardiovascular and Respiratory Variables

Cardiovascular and respiratory variables were recorded every ten minutes by a multiparameter anaesthesia monitor (GE Datex‐Ohmeda S/5 Compact Anästhesie Monitor, Germany): Heart rate (HR), respiratory rate (RR), ETCO2, inspired (FI ISO) and end‐tidal (ET ISO) isoflurane concentrations. The systolic, diastolic and mean arterial blood pressure (SAP, DAP, MAP respectively) were recorded by a second multiparameter monitor (Mindray, iPM12 Vet, China). This monitor also displayed the PPV. The MAP was already published as a global perfusion parameter for the concurrently conducted microperfusion study (Sauter et al. 2025). Furthermore, central venous pressure (CVP) was obtained. Cardiac output was measured during baseline (B), after the end of endotoxin infusion (ET0), 60 (ET60) and 120 (ET120) minutes after ending the endotoxin infusion, after fluid bolus (F) and after reaching normotension (MAP 80–90 mmHg) with NA infusion (NA).

### 2.6 Blood Samples

Blood sampling for arterial and mixed venous blood gas and for all jugular venous blood analysis was performed at fixed time points. During the instrumentation phase, measurements were taken 30 and 60 min after induction, and the two sets of values were averaged for baseline (B). Additional blood samples were taken at ET0, ET60, ET120, F, and NA. Blood gases were also taken before and five minutes after sedation, and for the analysis of endothelial glycocalyx components, an additional venous blood sample had been taken one day prior to the experiment in the awake horse.

Blood gas samples were anaerobically withdrawn in heparinised syringes and were analysed by a standard blood gas machine (AVL995, AVL Medizintechnik, Germany) for partial pressure of oxygen (PaO2 and PmvO2, respectively) and carbon dioxide (PaCO2 and PmvCO2, respectively), haemoglobin, lactate, and electrolytes.

Venous blood samples from the jugular catheter (10 mL) were transmitted into EDTA and serum tubes. Hematocrit (Ht) and leucocyte count (WBC) were measured (Automated Haematology Analyser XP‐300, Sysmex Deutschland GmbH; Germany), and a refractometer (RF.5612 clinical hand refractometer, Euromex Microscopen, Netherlands) was used to determine the total plasma protein content (TP). The serum tubes were allowed to clot for 20–30 min in an upright position. Then centrifugation was performed for 20 min at 3000 g followed by pipetting the serum. Samples were stored at ‐80°C until analysis with two specific validated ELISA kits for Syndecan‐1 (Horse Syndecan 1 (SDC1) ELISA Kit 48‐Strip‐Wells, Gentaur GmbH, Germany) and Heparan Sulphate Proteoglycan (Horse Heparan Sulphate Proteoglycan (HSPG) ELISA Kit 48‐Strip‐Wells, Gentaur GmbH, Germany), which were performed according to the manufacturer's instructions.

### 2.7 Trial End

**FIGURE 1:**
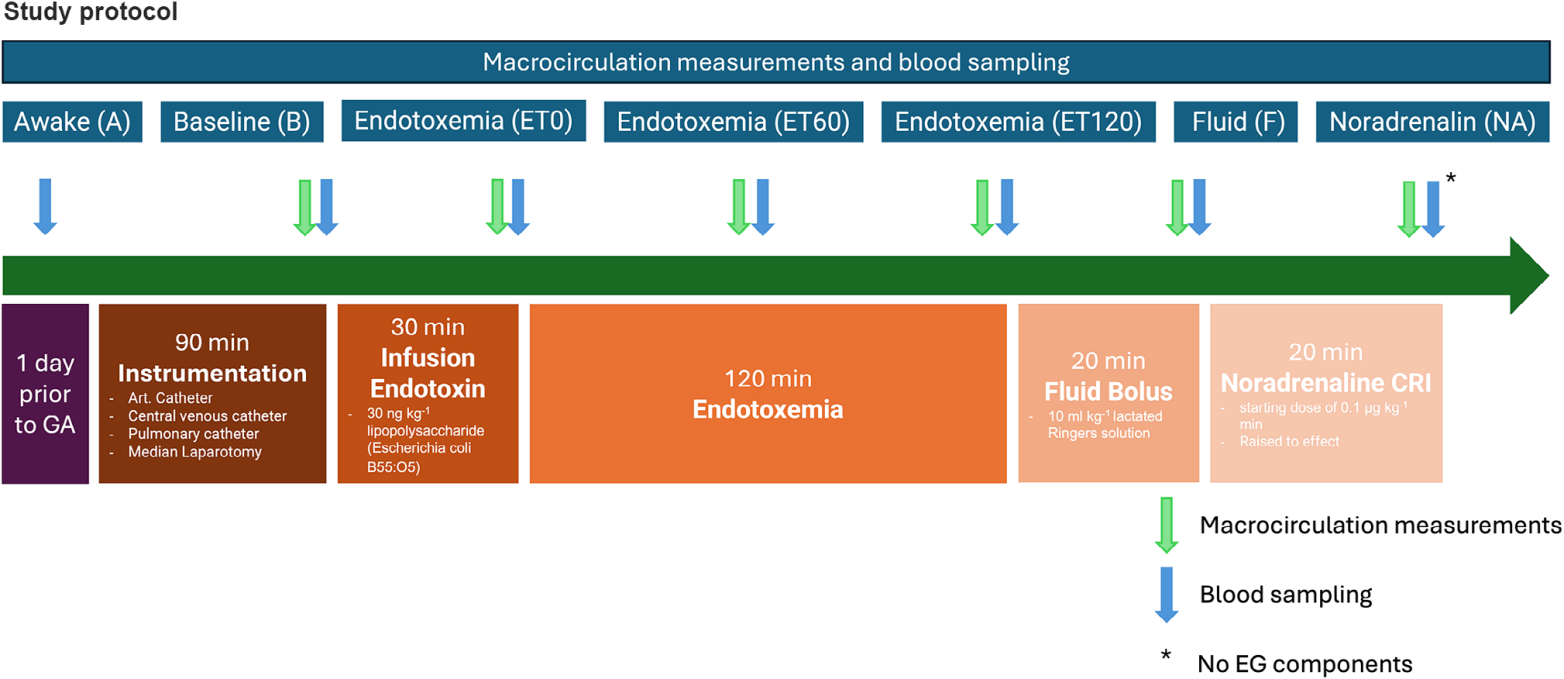
Graphical and time‐related representation of the experimental setup with actions. Venous blood samples were withdrawn from awake animals for endothelial glycocalyx shedding products analysis. During anaesthesia an instrumentation phase and baseline (B) determination were followed by an endotoxaemia phase (ET) after the endotoxin infusion. Further measurements were performed after injecting a fluid bolus (F) of 10 mL kg^−1^ over 20 min and noradrenaline at 0.1 to 0.8 µg kg‐1 min‐1 (NA). Cardiovascular, respiratory, and haematologic variables were assessed, and arterial, mixed venous, and venous blood samples were collected. Art., arterial; CO, cardiac output; EG, endothelial glycocalyx.

Horses were euthanised at the end of the experiment under general anaesthesia without regaining consciousness using pentobarbital 60 mg kg^−1^ IV (Figure 1).

### 2.8 Calculated Variables

A simplified formula was used to calculate the systemic vascular resistance index (SVRI) (Skimming et al. 1997) and cardiac index (CI) CI = cardiac output / kg, stroke volume index (SV), alveolar‐arterial oxygen difference (P(A‐a)O_2_), arterial and mixed venous oxygen content (CaO_2_, CmvO_2_), oxygen consumption (VO_2_I) and delivery index (DO_2_I), oxygen extraction ratio (ERO_2_), and shunt fraction were determined by standard equations (see Supplement).

### 2.9 Statistical Analysis

Results were logged using Excel (Excel for Mac, 2011, Microsoft, state, United States of America). The present data was analysed using SAS software (version 9.4M7) and SAS Enterprise Guide (Version 7.1, SAS Institute, United States of America). A significance level of 0.05 was applied. Graphical results representation was performed using GraphPad Prism 9 statistical software (GraphPad Software, Inc., US). The data distribution was visually validated using boxplots and the distributions of the residuals on quantile‐quantile plots. Descriptive statistics used median, minimum, maximum, mean, and standard deviation dependent on distribution. Parametric data were analysed using a linear repeated measures model with repeated measurements over time. The variance‐covariance structure was modelled using a heterogeneous autoregressive model, which allows for different variances and correlation between the time points. Subsequently, when a statistically significant difference between the measurement time points was presented, pairwise comparisons were calculated using the Tukey‐Kramer adjustment. The pairwise comparisons are presented between the baseline and the individual time point and between ET120 and F and NA.

## 3 Results

### 3.1 Animals and Noradrenaline Infusion

The median body weight was 581 kg (495–635) kg. The mean age of the animals was 12 years ± 5 years. All horses completed the study. Horses needed a median rate of NA of 0.46 (0.4 and 0.6, µg kg^−1^ min^−1^ (median; min and max).

### 3.2 Cardiovascular and Oxygenation Variables

**FIGURE 2:**
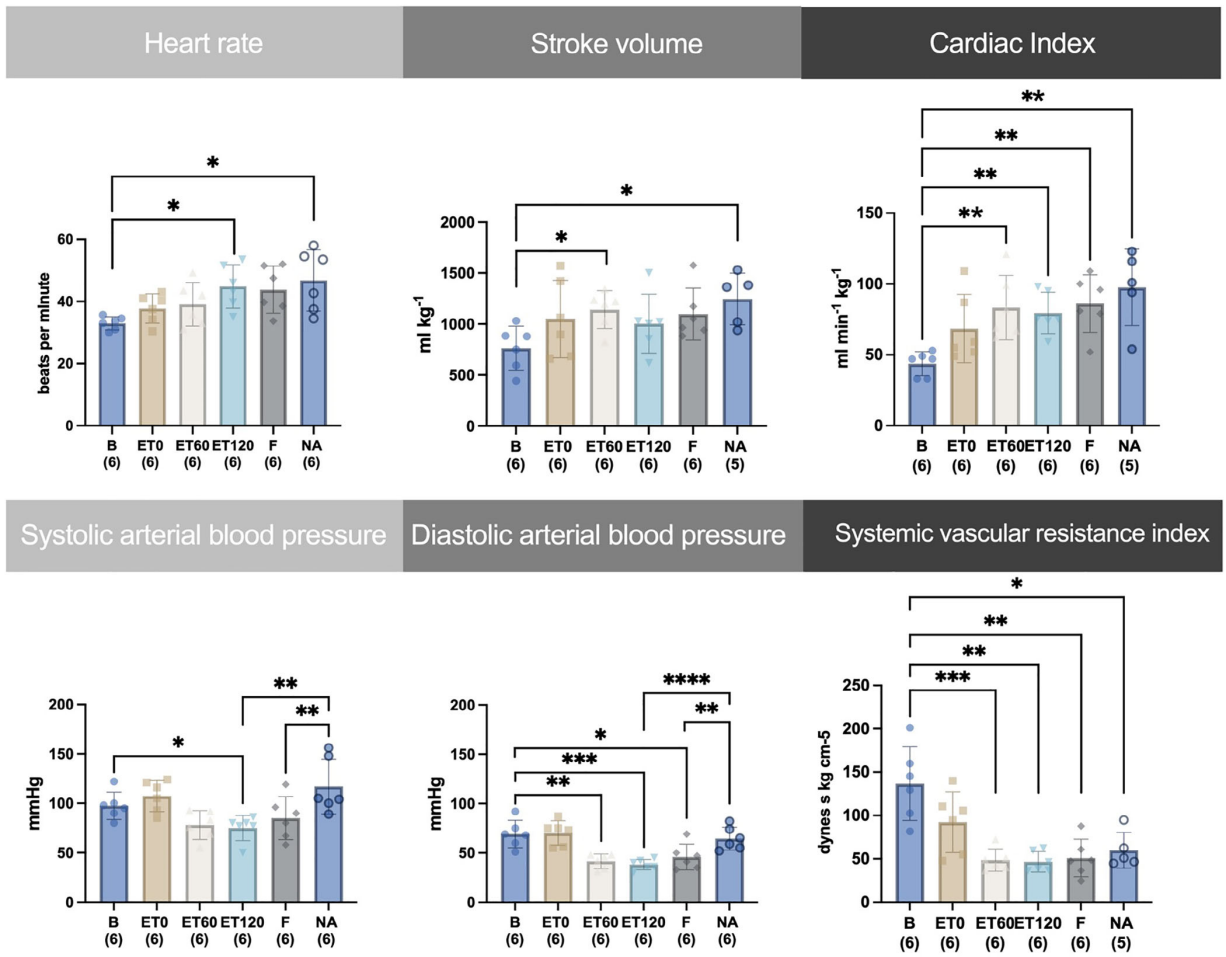
Time course of individual values, mean and standard deviation of heart rate, stroke volume, cardiac index, systolic and diastolic arterial blood pressure, and systemic vascular resistance index from six horses before (B) and after intravenously induced endotoxaemia (ET0, ET60, ET120), fluid bolus (F) and noradrenaline infusion (NA) under general anaesthesia with isoflurane. Data are presented as mean (bar) and standard deviation (whiskers). The numbers in brackets represent the number of subjects with successful measurements. The dots represent measurements from individual horses. Data were analysed using a linear mixed model and post‐hoc test with Tukey‐Kramer adjustment. A statistically significant difference is marked with asterisks (* = *p* < 0.05; ** = *p* < 0.01; *** = *p* < 0.001).

Cardiovascular values are presented in Figure 2. The HR was significantly increased compared to B at ET120 by 38 %, and at NA by 40 %. Stroke volume significantly increased after B at ET60 by 50 % and at NA by 63 %. Compared to B, CI significantly increased by 90 %, 82 %, 97 % and by 123 % at ET60, ET120, F and NA, respectively. The SAP decreased significantly compared to B at ET120 by 23 % and increased at NA compared to ET120 by 56 % and compared to F by 38 %. The DAP decreased significantly compared to B at ET60 by 40 %, at ET120 by 45 %, and at F by 34 % and increased at NA compared to ET120 by 79 % and to F by 50 %. The SVRI significantly decreased compared to B at ET60 by 65 %, at ET120 66 %, F by 64 % and at NA by 59 %. (Figure 2)

**FIGURE 3:**
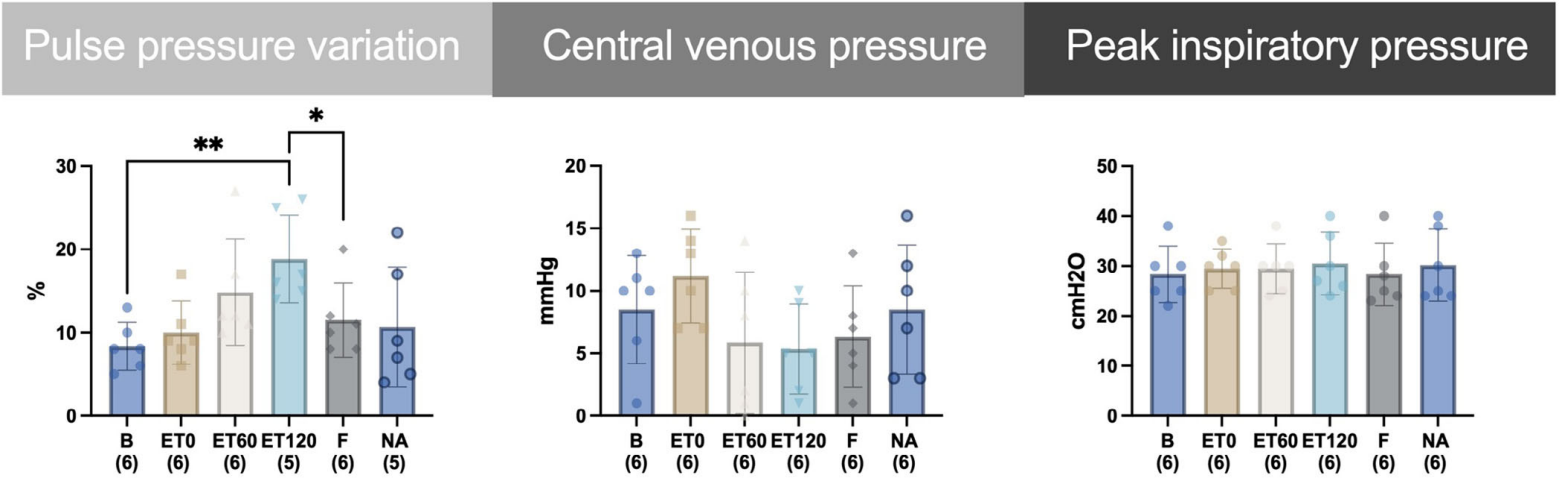
Time course of mean and standard deviation of pulse pressure variation, central venous pressure; peak inspiratory pressure from six horses before (B) and after intravenously induced endotoxaemia (ET0, ET60, ET120), fluid bolus (F) and noradrenaline infusion (NA) under general anaesthesia with isoflurane. Data are presented as mean (bar) and standard deviation (whiskers). The numbers in brackets represent the number of subjects with successful measurements. The dots represent measurements from individual horses. Data were analysed using a linear mixed model and post‐hoc test with Tukey‐Kramer adjustment. A statistically significant difference is marked with an asterisks (* = *p* < 0.05; ** = *p* < 0.01; *** = *p* < 0.001).

The PPV significantly increased compared to B at ET120 by 109 %. A significant reduction in PPV was seen after fluid administration for F compared to ET120 by 34 %. (see Figure 3). After the single fluid bolus, SV increased at F by 9.4 % compared to ET120.

**FIGURE 4:**
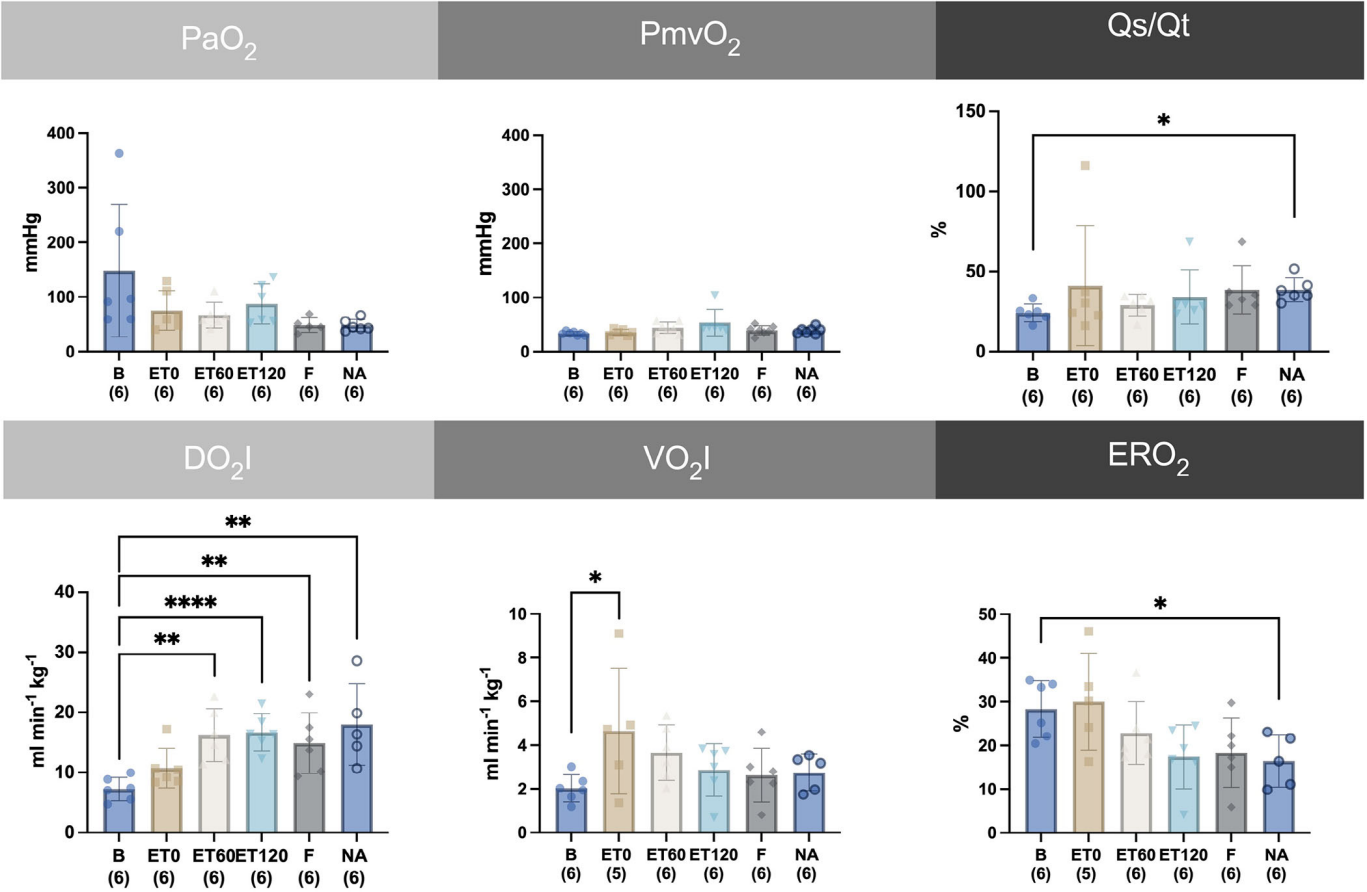
Time course of individual, mean and standard deviation of arterial partial pressure of oxygen (PaO_2_), mixed venous partial pressure of oxygen (PmvO_2_), shunt fraction (Qs/Qt), oxygen delivery index (DO_2_I), oxygen consumption index (VO_2_I), oxygen extraction ratio (ERO_2_) and from six horses before (B) and after intravenously induced endotoxaemia (ET0, ET60, ET120), fluid bolus (F) and noradrenaline infusion (NA) under general anaesthesia with isoflurane Data are presented as mean (bar) and standard deviation (whiskers). The numbers in brackets represent the number of subjects with successful measurements. The dots represent measurements from individual horses. Data were analysed using a linear mixed model and post‐hoc test with Tukey‐Kramer adjustment. A statistically significant difference is marked with asterisks (* = *p* < 0.05; ** = *p* < 0.01; *** = *p* < 0.001; **** = *p* < 0.0001).

The oxygenation variables are depicted in Figure 4. The FiO_2_ for all horses was 95 ± 1 %. There was no change in PaO_2_, PmvO_2_ compared to B. The shunt fraction Qs/Qt was significantly increased from B by 60 % for time point NA. Horses had a significantly increased oxygen delivery index compared to B at ET60 by 125 %, at ET120 by 132 %, at F by 106 % and NA by 150 %. Changes in oxygen consumption index were significant compared to B at ET0 by 90 %. The oxygen extraction ratio was significantly lower by 42 % at NA compared to B (see Figure 3).

**FIGURE 5:**
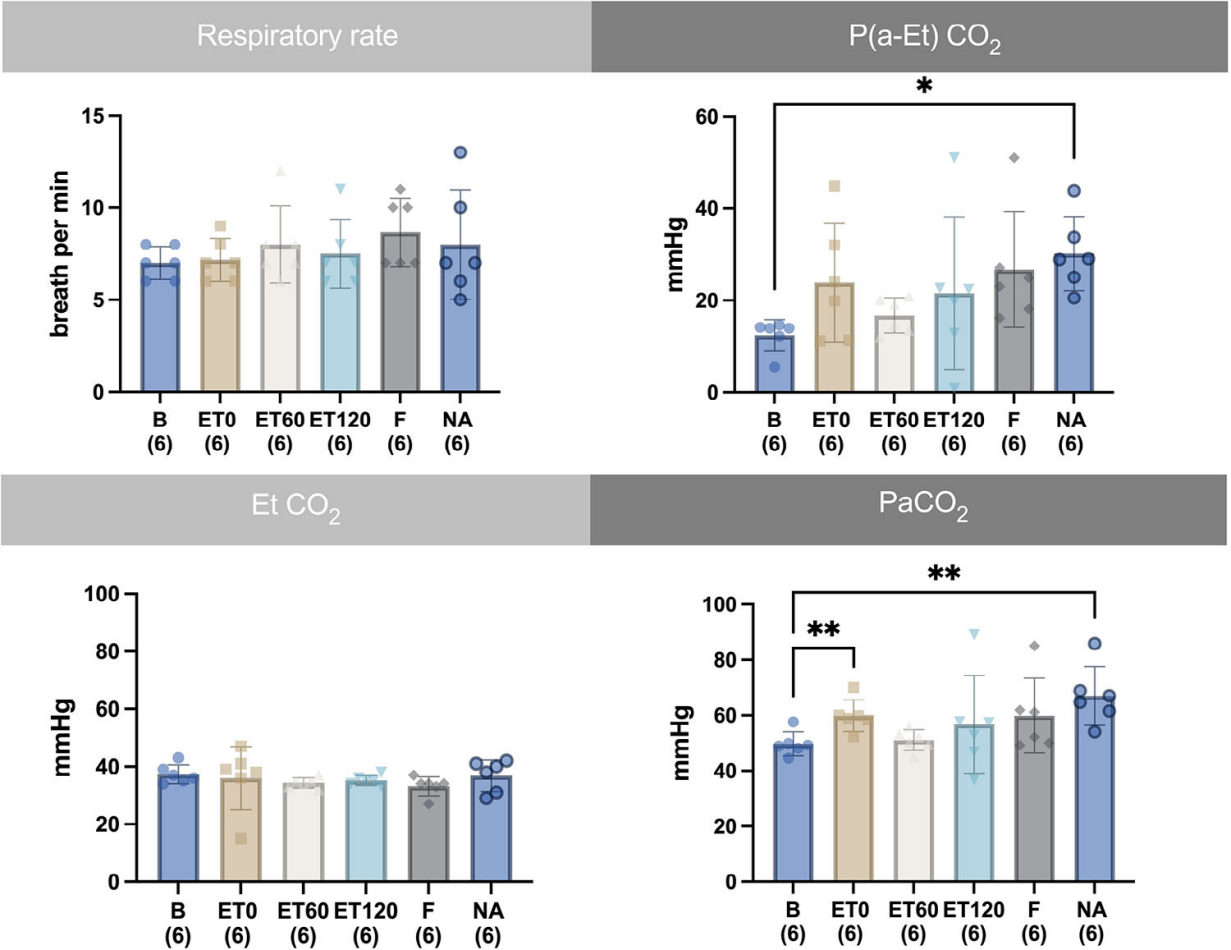
Time course of mean and standard deviation of respiratory rate, end‐tidal partial pressure of carbon dioxide (EtCO_2_), arterial to end‐tidal difference of carbon dioxide partial pressure (P(a‐Et)CO_2_), end‐tidal carbon dioxide concentration (Et CO_2_) arterial partial pressure of carbon dioxide (PaCO_2_) from six horses before (B) and after intravenously induced endotoxaemia (ET0, ET60, ET120), fluid bolus (F) and noradrenaline infusion (NA) under general anaesthesia with isoflurane. Data are presented as mean (bar) and standard deviation (whiskers). The numbers in brackets represent the number of subjects with successful measurements. The dots represent measurements from individual horses. Data were analysed using a linear mixed model and post‐hoc test with Tukey‐Kramer adjustment. A statistically significant difference is marked with asterisks (* = *p* < 0.05; ** = *p* < 0.01).

The respiratory variables are depicted in Figure 5. There was no significant change in RR and EtCO_2_. The P(a‐Et)CO_2_ was significantly higher compared to B for NA by 143 % and the PaCO_2_ was significantly higher at NA by 34 % compared to B. The pairwise comparisons are depicted in the graph (see Figure 5).

### 3.3 Blood Analysis

There was no significant difference in plasma concentration of SYN‐1 and HEP, respectively, at any point in time. (see Figure 5)

**FIGURE 6:**
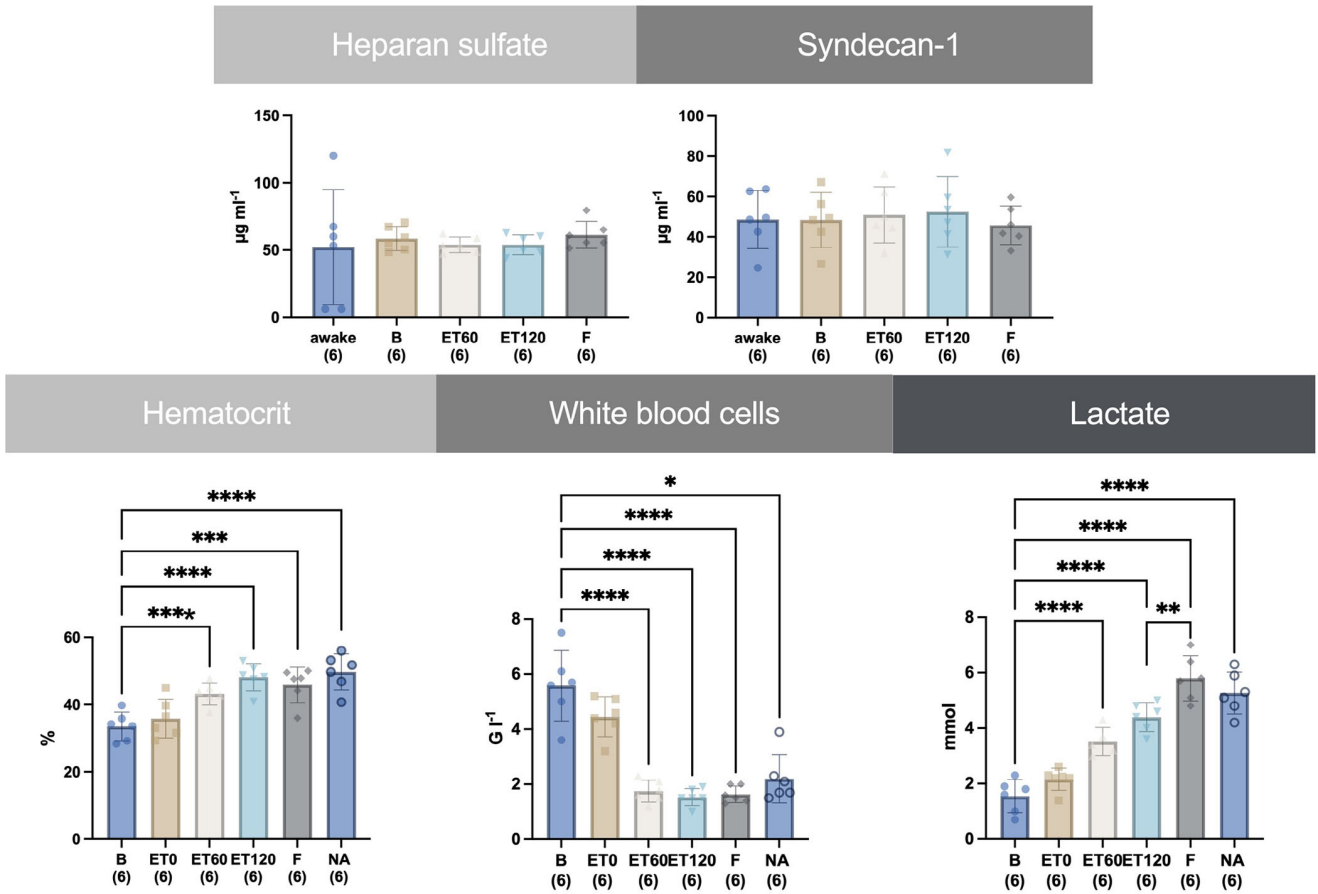
Time course of the mean and standard deviation of blood lactate concentration, white blood cells count and haematocrit from six horses before (B) and after intravenously induced endotoxaemia (ET0, ET60, ET120), fluid bolus (F) and noradrenaline infusion (NA) under general anaesthesia with isoflurane. Glycocalyx shedding products heparan sulphate and syndecan‐1 are presented as mean and standard deviation from six horses before anaesthesia (awake), before (B) and after intravenously induced endotoxaemia (ET60, ET120). Data are presented as mean (bar) and standard deviation (whiskers). The numbers in brackets represent the number of subjects with successful measurements. The dots represent measurements from individual horses. Data were analysed using a linear mixed model and post‐hoc test with Tukey‐Kramer adjustment. A statistically significant difference to B is marked with one or two asterisks (* = *p* < 0.05; ** = *p* < 0.01; *** = *p* < 0.001; **** = *p* < 0.0001).

The Ht was significantly increased from B to ET60 by 28 %, for ET120 by 43 %, for F by 37 % and for NA by 48 %. The white blood cells significantly decreased from B to ET60 by 69 %, for ET120 by 73 %, for F by 71 % and for NA 75 %. The lactate concentration was significantly increased compared to B at ET60 by 115 %, at ET120 by 180 %, at F by 289 % and NA by 248 %. (see   Figure 6)

## 4 Discussion

The current study revealed typical and well‐described early hyperdynamic changes in cardiovascular parameters with concurrent hypotension, increased PPV, leucopenia and hyperlactatemia consistent with acute endotoxaemia. However, no consistent increase of glycocalyx‐shedding products could be shown during this approximately 2.5 h of endotoxaemia.

The current study showed a significant increase in HR after endotoxin infusion, as observed in awake horses (Jacobs et al. 2013), as a clinical sign of endotoxaemia. The low SVRI at the onset of sepsis can cause a reflex tachycardia to maintain and even increase CI (Burrows 1970). We saw both an early and severe decrease of SVRI and a doubling of the CI. In part, these changes result from an endogenous catecholamine release, probably due to underperfusion of tissues, which activates cardiac β_1_‐adrenergic receptors, leading to increases in chronotropy (HR) and contractility (SV) (Motiejunaite et al. 2021). In the current study both the CI and the SV were stable or increased during endotoxaemia. The horses had a low CI with 43 ± 9 mL kg^−1^ min^−1^, while the MAP was stable before administration of endotoxin (B). It is well known that inhalant anaesthetics and positioning the horses in dorsal recumbency alone can negatively influence the contractility and therefore reduce cardiac output and MAP (Gasthuys et al. 1991; Blissitt et al. 2008). Therefore, dobutamine was used in the current study to compensate for the anaesthesia‐ and position‐induced drop in blood pressure. However, during the baseline measurement, the CI was not adequate.

The significant reduction in DAP 60 min and SAP 120 min after the end of endotoxin infusion was most likely caused by endotoxin‐induced changes in vascular smooth muscle reactivity. The vasodilation was reflected already at ET0 by a pronounced decrease in SVRI. At ET0 the horses were still able to maintain their blood pressure by increasing their CI. Another 60 min later, the mean DAP had fallen to < 40 mmHg and after 120 min, the SAP had decreased to < 90 mmHg, which highlights the failure of compensating mechanisms to maintain normotension. Current investigations suggest that vascular hyporeactivity in sepsis is caused by disruption of the regulation of vascular smooth muscle by certain vasoconstrictors. Rather than induced by general damage to smooth muscle cells or by endothelium‐dependent vasodilation (Zhang et al. 2014).

Pronounced vasodilation can result in relative hypovolemia, which is treated with crystalloid volume substitution to optimise blood pressure and oxygen delivery. In our experiment, the fluid bolus administration 120 min later did not result in any significant change of SAP or DAP. Possible reasons are endothelial dysfunction, capillary leak syndrome, and persistent vasodilation consistent with the still significantly decreased SVRI, which can compromise the effectiveness of fluid resuscitation efforts (Incalza et al. 2018). Another reason would be an insufficient fluid volume. A fluid bolus of 5 ‐ 10 mL kg^−1^ over 5 ‐ 10 min should induce an increase of SV of 10 ‐ 15 %, to be judged as a positive fluid response. In the current study only a mean increase of SV of 9.4 % could be seen with a fluid bolus of 10 mL kg^−1^ over 20 min. This does not exactly meet the criteria defined before but can probably be explained by the slower administration of the fluid bolus in horses limited by infusion pump rates and by the ongoing severe vasodilation. The PPV, as a dynamic, non‐invasive predictor of fluid responsiveness, exceeded 15 % at ET120, indicating a fluid‐responsive state. The administration of a 10 mL kg^−1^ bolus of Ringer's lactate significantly reduced the PPV to below 10%, suggesting an improved venous return with less impact of the artificial ventilation on the haemodynamic status. The discrepancy between PPV changes and stroke volume increases may reflect the complex cardiovascular effects of endotoxaemia, including alterations in vascular tone and myocardial function. Additionally, the ongoing vasodilation was probably too severe to show any beneficial effect on blood pressure after fluid administration. It has been shown previously that fluid responsiveness is often decreased during endotoxaemia and sepsis (Muehlestein et al. 2021) and that fluids alone cannot correct the tissue hypoxia and vasopressors are necessary (Evans et al. 2021). This theory is also supported by the fact that noradrenaline at a rate of 0.46 µg kg^−1^ min^−1^ could significantly improve SAP and DAP without decreasing CI and SV (Dancker et al. 2018). Noradrenaline activates α‐ and β‐ adrenergic receptors dose dependently. The alpha‐adrenergic effect results in the contraction of resistance and capacitance vessels. This sympathetic stimulation by promoting potent vasoconstriction was able to counteract the effects of endotoxaemia on the measured parameters in our study in a clinically used dose range. Additionally, stimulation of beta‐1 adrenergic receptors might have influenced the HR, which was observed in this study. Furthermore, while PPV thresholds have been validated in other species, their application in horses requires cautious interpretation, as equine cardiovascular physiology and endotoxemic responses may introduce unique variables. Future studies should evaluate the relationship between PPV, stroke volume changes, and other haemodynamic parameters in endotoxemic conditions to refine its clinical utility in equine critical care. Furthermore, the tidal volume should be kept constant and be reported during studies evaluating PPV.

The present study showed a non‐significant increase in oxygen consumption, while oxygen delivery was increased significantly after endotoxin. An increase in oxygen consumption may indicate a developing septic state (Rackow et al. 1988) and/or the increased oxygen demand of the heart due to a hyperdynamic state. The increased oxygen delivery is probably attributable to the increased CI and Ht. Physiologically, oxygen delivery is much higher than oxygen consumption, and only around 25 % of O_2_ is used, a value represented by the oxygen extraction ratio. This functional reserve allows for compensation of variations in regional oxygen demand. The local oxygen consumption can be increased without the need for an increased oxygen delivery (Hubbell and Muir 2015). Increased CI due to a rise in HR and SV together with an increased Ht resulted in a consecutive increase of oxygen delivery. The baseline oxygen extraction ratio of 23 ± 6 % of the present study is consistent with studies in humans (Beck et al. 2006) and horses (Benmansour et al. 2014). A continuous decreasing trend (becoming significant at NA) in oxygen extraction ratio was observed in the studied horses. This signifies that oxygen delivery was more increased than oxygen consumption. In the current study oxygen consumption was relatively low at B, which could be explained by the decreased metabolic rate due to sedatives and hypnotics (Edner et al. 2007). During sepsis oxygen consumption can be further decreased by increased shunt formation in endotoxic, septic processes, but such a state was never reached in the current study, and endotoxaemia rather increased oxygen consumption (not significant) (Edner et al. 2007). Sepsis and impaired cellular oxygen utilisation may also decrease the oxygen consumption and therefore, oxygen extraction rate (Park et al. 2015). Mechanisms of cytopathic hypoxia describe the inability of tissues to metabolise oxygen adequately. There is increased NO synthesis in sepsis due to mitochondrial dysfunction (Fink 2002). Nitric oxide inhibits the mitochondrial respiratory chain (Borutaité and Brown 1996). However, in the current study, no reduction of oxygen consumption was observed, and the reduction of oxygen extraction ratio was caused by the significantly increased oxygen delivery. Human studies have observed an association of low oxygen extraction rate with multiple organ failure and higher mortality rate in patients with severe sepsis or in septic shock (Park et al. 2015).

During the experiment, PaO_2_ values lower than 60 mmHg were measured, which meets the definition of hypoxaemia. The PaO_2_ is influenced by the diffusion capacity of alveolar capillaries, inspiratory oxygen fraction, alveolar ventilation, and ventilation‐perfusion ratio. General anaesthesia and dorsal recumbency alone can cause a decrease in PaO_2_ due to decreased residual capacity (Hall et al. 1968). Perfusion without ventilation provides an increase in the intrapulmonary right‐to‐left shunt. After infusion of endotoxin, the shunt fraction increase became only statistically significant at NA. Statistical significance was reached at the time point, probably because the SD was decreased. There was a trend of higher shunt fractions throughout the study. Clinically significant shunt formation under general anaesthesia is thought to occur above a shunt fraction of 20 % to 30 % (Nyman and Hedenstierna 1989). Due to microcirculatory dysfunction, pulmonary shunt enhancement may occur in endotoxemic, septic processes (Ellis et al. 2002; Ince 2005). However, in the current study, no significant changes could be seen induced by endotoxin alone. We are not aware of any reason why the addition of NA should increase the shunt fraction during endotoxaemia and believe it is only coincidental that statistical significance was reached after NA.

In the current study, lactate levels increased before a significant drop in blood pressure was observed. Elevated lactate concentrations are often an indicator of systemic tissue hypoperfusion and cellular dysfunction (Singer et al. 2016). However, impaired tissue oxygenation is likely not the sole cause of hyperlactatemia in sepsis. Reduced global blood flow and inadequate distribution can lead to decreased liver perfusion, which impairs lactate clearance and consequently raises blood lactate levels. In this study, horses had an increase in the heterogeneity index after endotoxin application (Sauter et al. 2025). Additionally, a drop in blood pressure can further compromise tissue oxygenation in horses (Hopster et al. 2015). The primary cause of hyperlactatemia in horses is thought to be an anaerobic metabolic state caused by insufficient oxygen supply. Other contributing factors include increased sodium/potassium ATPase activity due to inflammatory mediators, inhibition of pyruvate dehydrogenase in glucose metabolism, and elevated lactate production by inflammatory cells. During sepsis, the inactivation of pyruvate dehydrogenase leads to higher levels of lactate and pyruvate. A combination of these mechanisms is likely responsible for the observed hyperlactataemia in this case.

In the current study, a highly significant leukocyte drop with subsequent leukocytopenia was observed, similar to those ones observed after endotoxin infusion with intravenous (Jacobs et al. 2013) and intraperitoneal (Peiró et al. 2010) administration of bacterial lipopolysaccharides. Endotoxins cause increased leucocyte adherence, which can lead to leukopenia. In addition, endothelial glycocalyx degradation itself would increase leucocyte cell adhesion (Vink et al. 2000), even though we were not able to detect a degradation. A possible involvement of proinflammatory cytokines, specifically TNFα (Peiró et al. 2010) in the leukopenic response of horses has been described.

In this study, no evidence of endothelial glycocalyx shedding was found. The horse accounts for an LPS‐sensitive mammalian species (MacKay et al. 1991) and therefore we believed that degradation of EG would be measurable at an endotoxin's 30 ng kg^−1^ body weight. Recent studies show increased plasma syndecan‐1 concentration in 25 % of the horses classified as septic (Hobbs et al. 2023). This classification was based on human reference values (0 ‐ 40 ng ml^−1^) (Wei et al. 2018). This classification seems to make little sense since the values in humans and horses seem to differ significantly in terms of the unit of measurement. The reference values given refer to nanograms, while the syndecan‐1 values measured in horses are in the microgram range. The average syndecan‐1 level in healthy horses was first reported as 16 ± 11 µg ml^−1^ (mean and SD). Our baseline values were higher than formerly reported values. A possible reason for the different baseline values can be seen in the different study population, and different ELISA kits. Insufficient shedding of degradation products may have contributed to the non‐significant results. This may be explained by a low endotoxin dose and a milder disease course in the present study, than in the clinical patient with sepsis. However, also in the formerly referenced study only 25 % of the horses had increased syndecan‐1 values. In sepsis, increased secretion of proinflammatory mediators and the presence of oxygen radicals can activate proteases, leading to the enzymatic breakdown of glycocalyx components (Mulivor and Lipowsky 2004; Yini et al. 2015; van Golen et al. 2012). Factors such as heightened microvascular permeability and excessive immune system activation may contribute to this process. A strong link between lipopolysaccharide‐induced sepsis, glycocalyx degradation, and elevated biomarkers like syndecan‐1 has been identified in humans (Anand et al. 2016; Saoraya et al. 2021), as well as in animal models involving dogs (Yini et al. 2015) and laboratory animals (Iba et al. 2020). In dogs, glycocalyx breakdown, indicated by shedding products such as syndecan‐1 and heparan sulphate, correlates with higher levels of inflammatory mediators like IL‐6 and TNFα during septic shock. (Yini et al. 2015). In human studies, the infusion of 1 ng kg^−1^ body weight of endotoxin was shown to reduce endothelial glycocalyx thickness by 50 % (Nieuwdorp et al. 2008). The measured basal syndecan‐1 and heparan sulphate concentrations in horses are approximately 1000 times higher than in dogs (Yini et al. 2015) and humans (Anand et al. 2016, Saoraya et al. 2021) and 10 times higher than in pigs (Hofmann‐Kiefer et al. 2009). It could therefore be possible that potential increases caused by shedding are more difficult to detect. This contrasts with studies on horses in which septic patients saw a significant increase (Hobbs et al. 2023). Nonetheless, the different basal values between species emphasised the need to introduce species‐specific reference values.

Experimental endotoxaemia of the horse is widely established. A dosage of 30 ng kg^−1^ body weight (Jacobs et al. 2013, Cudmore et al. 2013; Morris et al. 1990; Moore et al. 2007) or 50 ng kg^−1^ body weight (Allen et al. 1996) administered intravenously is normally used to induce endotoxaemia. Different dosages of bacterial endotoxin in different animal species are described to induce endotoxaemia with dosages up to 10 mg kg^−1^ in pigs (Hofmann‐Kiefer et al. 2009). It is also possible that the early time of the disease process after the endotoxin infusion may play a role. Studies show increased plasma concentration can often only be expected after several hours to 2 days (Hofmann‐Kiefer et al. 2009; Shaw et al. 2021). In naturally parvovirus‐infected dogs, syndecan‐1, heparan and sulphate were not significantly elevated at hospital admission (Naseri et al. 2020).

Degradation of endothelial glycocalyx has also been associated with IV administration of fluids in human patients (Hippensteel et al. 2019) In a haemorrhagic shock model in dogs, large volumes of crystalloids were associated with higher hyaluronan values, indicating potential glycocalyx shedding (Smart et al. 2018) The study situation on the influence of IV fluid therapy on the shedding of individual glycocalyx degradation products is controversial (Naseri et al. 2020). A recent study has failed to demonstrate increased shedding (Macdonald et al. 2022). The latter is consistent with observations from the study conducted here. There wasn't a significant increase in the shedding components of the endothelial glycocalyx induced by fluid infusion.

The study's limitations include the small sample size and the lack of a placebo group. A larger population comparing healthy and diseased individuals could be beneficial. Anaesthesia and anaesthetic drugs, as well as a low endotoxin dose, might have ameliorated changes. A further limitation can be seen in the duration of the study, as the time frame for sampling shedding products might be too short to detect any elevations.

The low CI at B was not ideal as a starting point. During experimental studies the anaesthetist still relies on basic cardiovascular parameters such as HR and MAP, which were within normal limits. The current study shows that we are not aware of the current cardiovascular status of our patients when measurement of cardiac output is not available or not evaluated further, as it happened in the current study. In future studies, baseline measurements before administration of endotoxin could be standardised further.

## 5 Conclusion

In summary, intravenous infusion of endotoxin induced a hyperdynamic and hypotensive cardiovascular status with typical changes in lactate, WBC, Ht and oxygenation parameters. The PPV might be a sensitive indicator for initiating fluid therapy; the inability to achieve the stroke volume criterion underscores the need for multimodal assessment of fluid responsiveness. The amount of endotoxin used for this study did not induce significant degradation of the endothelial glycocalyx within 3–4 h, as measured by syndecan‐1 and heparan sulphate plasma levels.

## Author Contributions

**Stephan Neudeck**: conceptualisation, investigation, writing ‐ original draft, methodology, validation, writing ‐ review and editing, data curation, supervision, project administration, formal analysis. **Philipp K. Sauter**: investigation, writing ‐ original draft, visualisation, data curation, formal analysis. **Annette P. N. Kutter**: investigation, writing ‐ review and editing, visualisation, resources. **Barbara Steblaj**: investigation, writing ‐ review and editing, visualisation, resources. **Franz J. Söbbeler**: investigation, conceptualisation, writing ‐ review and editing. **Julia Reiners**: investigation, writing ‐ review and editing. **Fritjof Freise**: formal analysis, software, writing ‐ review and editing. **Alvaro J. Gutiérrez Bautitsta**: investigation. **Sabine B. R. Kästner**: conceptualisation, investigation, writing ‐ review and editing, project administration, supervision, resources.

## Ethical Statement

Ethical approval was obtained from the Ethics Committee of the State of Lower Saxony, Germany (approval number 33.19‐42502‐04 ‐ 20/3481).

## Conflicts of Interest

The authors declare no conflicts of interest.

## Supporting information




**Supporting File**: vms370458‐sup‐0001‐SuppMat.docx

## Data Availability

The data that support the findings of this study are openly available in Mendeley at DOI: 10.17632/nxr88zty63.1
