# Supplementary material for: Effects of Induced Endotoxaemia on Global Cardiovascular, Oxygenation and Haematologic Variables and the Integrity of the Endothelial Glycocalyx in the Horse
Source: Vet Med Sci. 2025 Jun 19;11(4):e70458. doi: 10.1002/vms3.70458 (PMC13007552; doi:10.1002/vms3.70458)
Supplement: Supplementary file 1 — Supporting File: vms370458‐sup‐0001‐SuppMat.docx [file VMS3-11-e70458-s001.docx]

**Supplement**

Systemic vascular resistance index (SVR dynes s kg cm^-5^) = MAP x 80 / CI (Skimming et al., 1997)

Stroke volume index (SVI ml beat^-1^ kg^-1^) = CI / HR (Haskins et al., 2005)

Alveolar dead space (%) = (PaCO_2_-E_T_CO_2_) / PaCO_2_ (Severinghaus & Stupfel, 1957)

Alveolar-arterial oxygen difference (P_(A-a)_O_2_ mmHg) = [(PB-PH_2_O) * FiO_2_ – PACO_2_ / R] – PaO_2_

PB = barometric pressure; PH_2_O = water vapor partial pressure; PACO_2_ = alveolar carbon dioxide partial pressure; R = respiratory exchange ratio (0.8)

Arterial oxygen content (CaO_2_ml dl^-1^) = (Hb x 1.39 x SaO_2_) + 0.003 x PaO_2_ (Haskins et al., 2005)

Venous oxygen content (CvO_2_ml dl^-1^) = (Hb x 1.39 x SvO_2_) + 0.003 x PvO_2_ (Haskins et al., 2005)

Mixed venous oxygen content (CmvO_2_ml dl^-1^) = (Hb x 1.39 x 1) + 0.003 x PAO_2_ (Haskins et al., 2005)

Oxygen consumption index (VO_2_I ml min^-1^ kg^-1^) = (CaO2 – CmvO2) x (CI (kg)/100) (Haskins et al., 2005)

Oxygen delivery index (DO_2_I ml min^-1^ kg^-1^) = CaO_2_ x (CI (kg)/100) (Haskins et al., 2005)

Oxygen extraction ratio (ERO_2_ %) = (VO_2_ / DO_2_) (Haskins et al., 2005)

Shunt fraction (Q_S_/Q_T_ %) = (CcO_2_-CaO_2_) / (CcO_2_-CmvO_2_) x 100 (Haskins et al., 2005)

Hb = hemoglobin; 1.39 = volume of oxygen bound to 1 gram of saturated haemoglobin; SaO2 = percentage of haemoglobin fully saturated with oxygen; Cc = pulmonary end-capillary O_2_ content
